# Supplementary material for: TAp73 Inhibits EMT and Cell Migration in Pancreatic Cancer Cells through Promoting SMAD4 Expression and SMAD4-Dependent Inhibition of ERK Activation
Source: Cancers (Basel). 2023 Jul 26;15(15):3791. doi: 10.3390/cancers15153791 (PMC10417771; doi:10.3390/cancers15153791)
Supplement: Supplementary file 1 [file cancers-15-03791-s001.zip › cancers-2490508-supplementary.pdf]

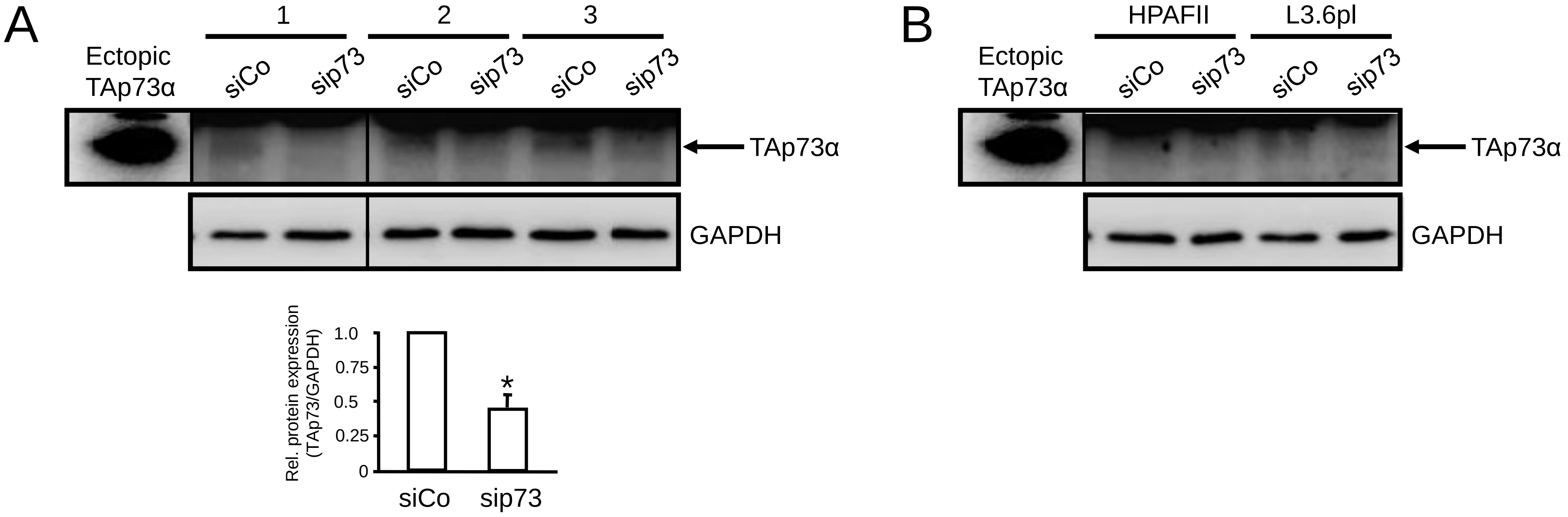

Figure S1. Verification of the RNA interference-mediated knockdown of TAp73 in PDAC-derived cell lines. (**A**) PANC-1 cells were transfected in three independent experiments (1-3) with 50 nM each of either control siRNA (siCo) or p73 siRNA (sip73) as outlined in the Methods section. Transfected cells were processed for immunoblotting of TAp73, and GAPDH as a loading control. PANC-1 cells which received an expression vector for TAp73*α* (ectopic TAp73*α*) were loaded side-by-side (left lane, blot under-exposed relative to the other lanes) to aid in identifying the band for the endogenous TAp73*α* isoform (arrow). A strong band of slightly lower electrophoretic mobility and unknown identity is partially overshadowing the band for TAp73*α* (see the uncropped version of this blot). The graph underneath the blot shows the results from densitometry-based quantification of band intensities (means ± SD, n = 3). The asterisk (∗) denotes a significant difference. (**B**) As in (A), except that protein lysates from siCo or sip73 transfected HPAFII or L3.6pl cells were used. The vertical lines indicate removal of irrelevant lanes.

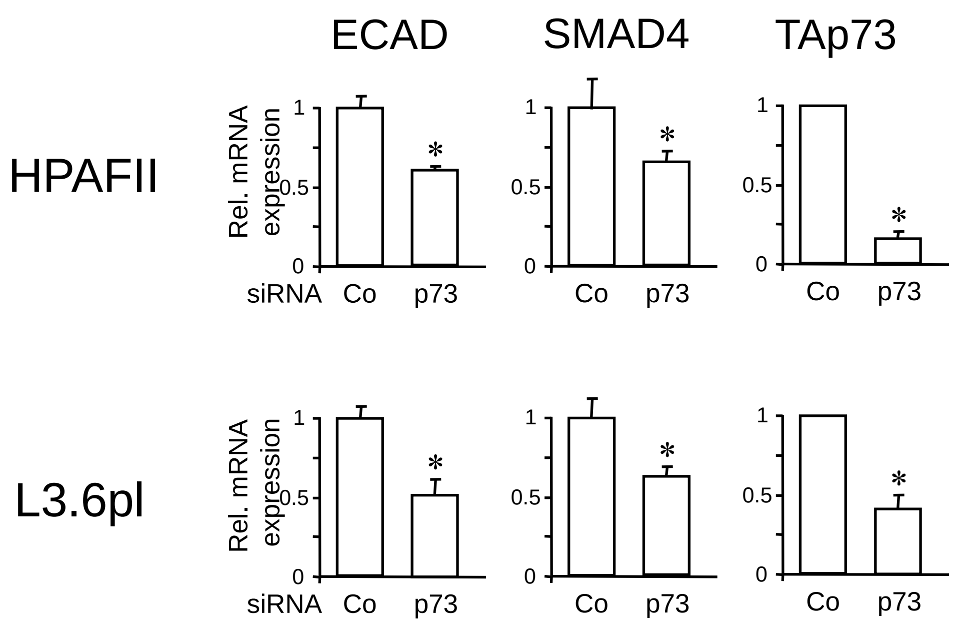

Figure S2. Effect of TAp73 knockdown on ECAD and SMAD4 expression in HPAFII and L3.6pl cells. HPAFII or L3.6pl cells were transfected with 50 nM each of either control (Co) siRNA or p73 siRNA as described in the Methods section. Forty-eight h later transfected cells were processed for qPCR analysis of ECAD and SMAD4, p73 for evaluation of successful knockdown, and GAPDH to control for small differences in RNA input. Data are the normalized mean ± SD of three experiments. The asterisks (*) indicate a significant difference ($p<0.05$).

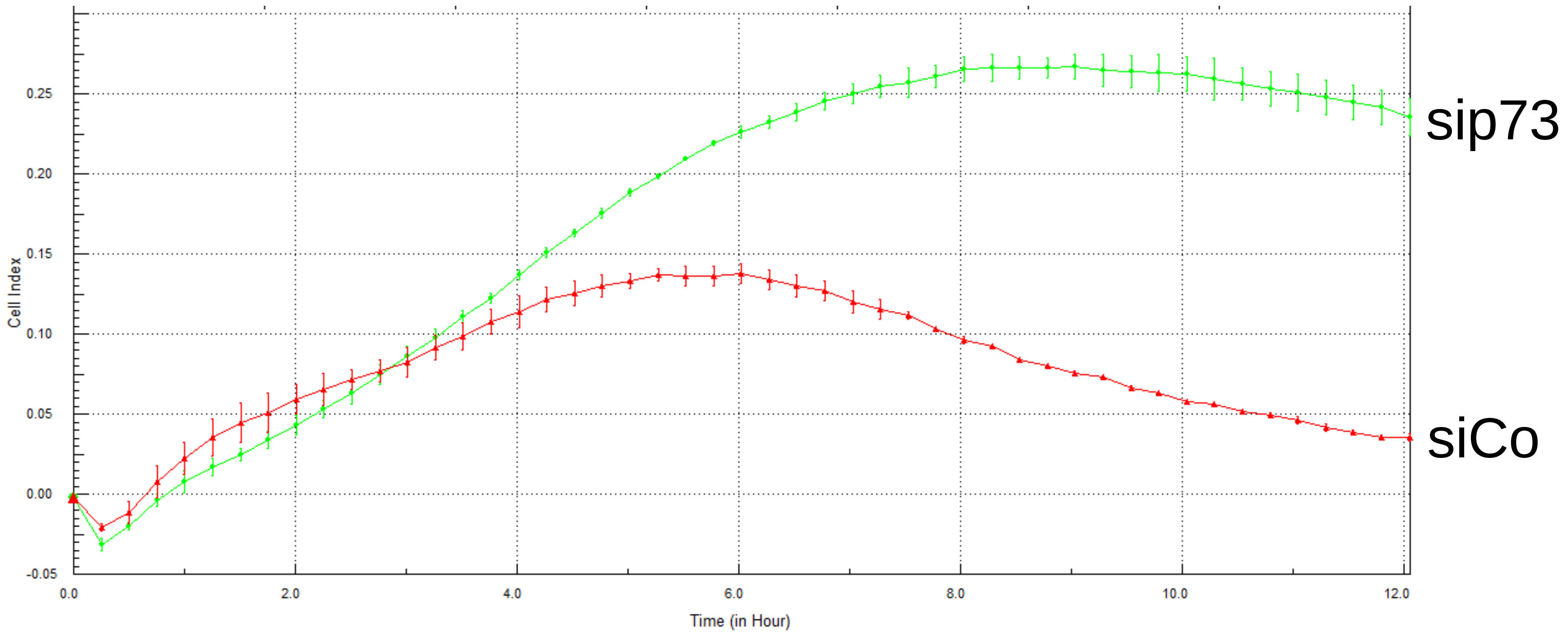

Figure S3. TAp73 inhibits cell migration in HPAFII cells. HPAFII cells were transiently transfected twice (on two consecutive days) with 50 nM of either control siRNA (siCo) or p73 siRNA (sip73) using RNAiMAX and subsequently subjected to cell migration assay on an xCELLigence platform. Measurements of basal migratory activity were taken every 60 min and graphically displayed as the dimensionless Cell Index plotted against assay time (in h). Data are from a representative experiment out of three experiments performed in total (means ± SD from 3-4 parallel wells).

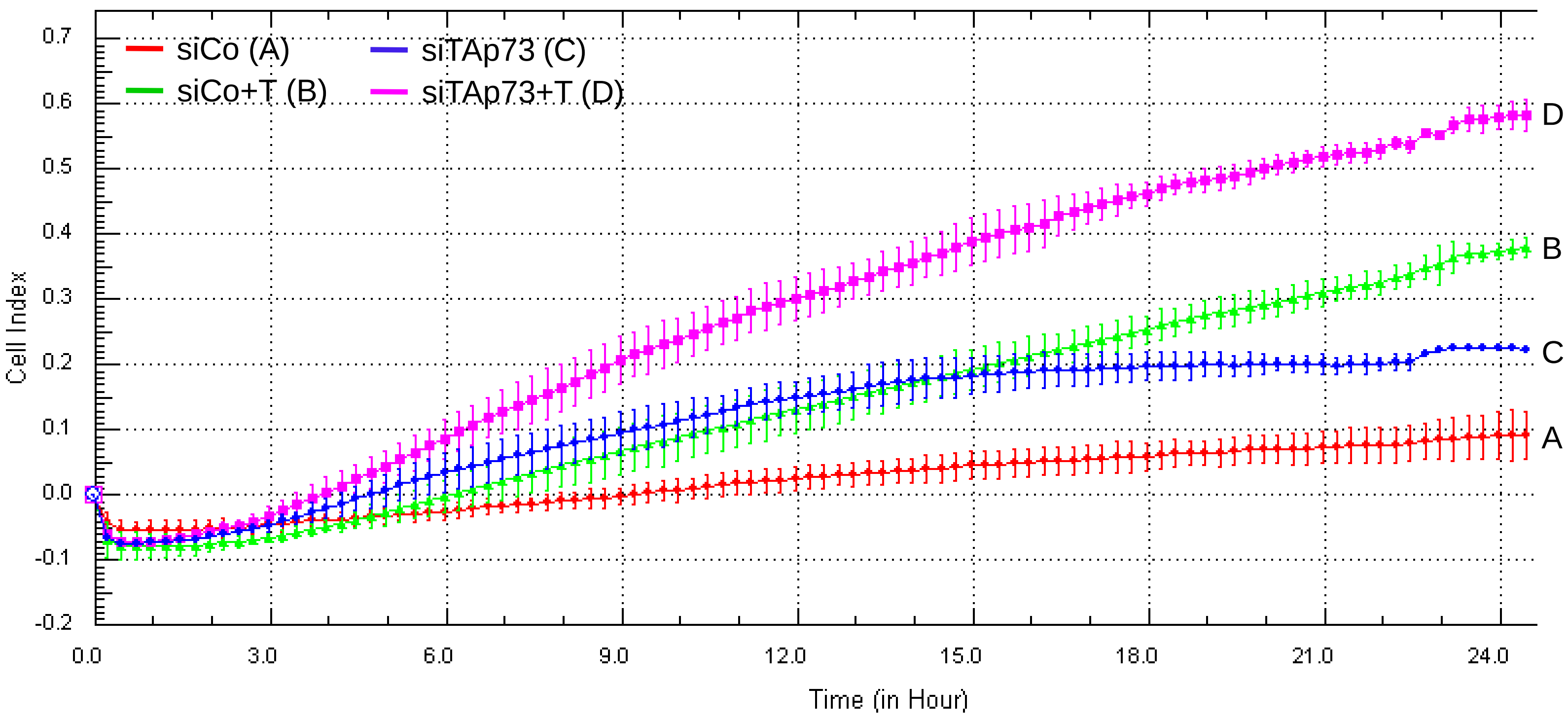

Figure S4. TAp73 inhibits basal and TGF-β-induced cell invasion in PANC-1 cells. PANC-1 cells were transiently transfected twice (on two consecutive days) with 50 nM of either control siRNA (siCo) or p73 siRNA (sip73) using Lipofectamine 2000. Subsequently, cells were subjected to cell invasion assay on an xCELLigence platform using Matrigel as barrier. During the assay one half of the cells was treated with 5 ng/ml TGF-β1 (+T), while the other half remained untreated. Measurements of invasive activity were taken every h and graphically displayed as the Cell Index plotted against assay time (in h). Data are from a representative experiment out of three experiments performed in total (means ± SD from 3 parallel wells).
